# Characterization of a GH43 Bifunctional Glycosidase from Endophytic *Chaetomium globosum* and Its Potential Application in the Biotransformation of Ginsenosides

**DOI:** 10.3390/biotech14010018

**Published:** 2025-03-12

**Authors:** Yao Lu, Qiang Jiang, Yamin Dong, Runzhen Ji, Yiwen Xiao, Du Zhu, Boliang Gao

**Affiliations:** 1 Key Laboratory of Natural Microbial Medicine Research of Jiangxi Province, Jiangxi Science and Technology Normal University, Nanchang 330013, China; 2 Key Laboratory of Microbial Resources and Metabolism of Nanchang City, Jiangxi Science and Technology Normal University, Nanchang 330013, China; 3 Jiangxi Province Key Laboratory of Biodiversity Conservation and Bioresource Utilization, Nanchang 330022, China

**Keywords:** α-L-arabinofuranosidase, β-D-xylosidase, biotransformation, endophytic fungi, GH43 family

## Abstract

The GH43 family of glycosidases represents an important class of industrial enzymes that are widely utilized across the food, pharmaceutical, and various other sectors. In this study, we identified a GH43 family glycoside hydrolytic enzyme, *Xyaf313*, derived from the plant endophytic fungus *Chaetomium globosum* DX-THS3, which is capable of transforming several common ginsenosides. The enzyme function analysis reveals that Xyaf313 exhibits dual functionality, displaying both α-L-arabinofuranosidase and β-D-xylosidase activity. When acting as an α-L-arabinofuranosidase, Xyaf313 achieves optimal enzyme activity of 23.96 U/mg at a temperature of 50 °C and a pH of 7. In contrast, its β-D-xylosidase activity results in a slight reduction in enzyme activity to 23.24 U/mg, with similar optimal temperature and pH conditions to those observed for the α-L-arabinofuranosidase activity. Furthermore, Xyaf313 demonstrates considerable resistance to most metal ions and common chemical reagents. Notably, while the maximum enzyme activity of Xyaf313 occurs at 50 °C, it maintains high activity at room temperature (30 °C), with relative enzyme activity exceeding 90%. Measurements of ginsenoside transformation show that Xyaf313 can convert common ginsenosides Rc, Rb_1_, Rb_2_, and Rb_3_ into Rd, underscoring its potential for pharmaceutical applications. Overall, our findings contribute to the identification of a new class of bifunctional GH43 glycoside hydrolases, highlight the significance of plant endophytic fungi as a promising resource for the screening of carbohydrate-decomposing enzymes, and present new candidate enzymes for the biotransformation of ginsenosides.

## 1. Introduction

Glycoside hydrolase 43 (GH43) is a significant component of carbohydrate hydrolases. The CAZy database (https://www.cazy.org/) currently includes over 50,000 genes encoding this class of enzymes. However, the functions and activity of the majority of the GH43 glycosidases remain largely unknown, with only 239 enzymes characterized to date (as shown in the CAZy database). This enzyme class is primarily derived from microorganisms, with a smaller proportion originating from plants, while no instances have been reported from animals (https://www.cazy.org/). The glycosidase activity associated with the GH43 family predominantly includes β-D-xylosidase (EC 3.2.1.37), α-L-arabinofuranosidase (EC 3.2.1.55), endo-α-L-arabinofuranosidase (EC 3.2.1.99), and 1,3-beta-galactosidase (EC 3.2.1.145) activity [1]. Numerous studies have demonstrated that GH43 glycosidases are significantly enriched in lignocellulose-degrading microorganisms and are closely associated with hemicellulose degradation [2,3,4]. Furthermore, glycosidases from the GH43 family are widely utilized as industrial enzymes, playing crucial roles in the preparation of oligosaccharide prebiotics [5], juice clarification [5], and pulp processing [5] and in the biofuel [6] and biopharmaceutical industries [5].

Natural saponins are the primary active compounds found in many medicinal plants, exhibiting a diverse spectrum of biological activity and promising applications [7]. Among these, ginsenosides represent a notable class of terpenoid saponins, recognized for their significant medicinal value and health-promoting functions, including immune regulation, hypoglycemic effects, anti-tumor properties, and anti-aging benefits [8]. However, the predominant PPD-type ginsenosides, such as ginsenosides Rb_1_, Rb_2_, and Rc, have limited absorption in the human intestine [8]. In contrast, low-glycosyl ginsenosides, including Rd, Rg_3_, and CK, which contain fewer sugar moieties, are more readily absorbed into the bloodstream and act as active compounds [9]. However, the complex sugar structures of ginsenosides pose challenges during chemical hydrolysis with acids or alkalis, resulting in a complex mixture of products and low yields of the desired compounds. This complexity often complicates subsequent separation processes and can lead to environmental pollution. Biotransformation, noted for its specificity, high catalytic efficiency, and environmental friendliness, is particularly well suited for the modification of complex saponins such as ginsenosides [8,10]. This method is widely employed for the production of rare ginsenosides, although it necessitates the use of efficient catalytic enzymes. The enzyme most commonly involved in the biotransformation of ginsenosides is β-glucosidase [10]. For example, the β-glucosidase Tsbgl, derived from *Thermoclostridium stercorarium*, can hydrolyze all outer glucose groups of protopanaxadiol-type ginsenosides, including Rb_1_, Rb_2_, Rc, and Rb_3_, as well as protopanaxatriol-type ginsenosides such as Re and Rg_1_, resulting in the production of various rare ginsenosides [11]. Upadhyaya et al. [12] reported the purification of a novel β-glucosidase (BGL) from *Armillaria mycelium*, which hydrolyzes ginsenoside Rc and can simultaneously cleave α-(1→6)-arabinofuranose at the C-20 position of the Rc bond or an external β-(1→2)-glycosidic bond at the C-3 position of Rc, yielding Rd and C-Mc1. Despite the diverse conversion pathways exhibited by these β-glucosidases, challenges such as complex product formation and low specificity remain prevalent [10]. As a result, the screening of enzymes with excellent selectivity continues to be a critical step in the current production of rare ginsenosides.

Recent studies have demonstrated that multiple alpha-L-arabinofuranosidases derived from microorganisms can effectively convert ginsenosides, leading to the production of various other ginsenosides. For example, the thermostable alpha-L-arabinofuranosidase from *Caldicellulosiruptor saccharolyticus* is capable of converting Re to Rd [13], while an alpha-L-arabinofuranosidase from the GH51 family of glycosidases can transform Rc into Rd [14]. Notably, many glycosidases within the GH43 family exhibit alpha-L-arabinofuranosidase activity; however, to our knowledge, the capacity of this class of enzymes to convert ginsenosides remains largely unexplored. Simultaneously, GH43 and β-glucosidase work synergistically to hydrolyze ginsenosides, thereby facilitating the preparation of rare ginsenosides containing one to two sugar units. Furthermore, some GH43 glycosidases display multifunctional activity, which could reduce enzyme usage and minimize redundant operational processes during ginsenoside conversion [15,16]. In summary, GH43 glycosidases present significant potential for the biotransformation of saponin products, such as ginsenosides, owing to their ability to hydrolyze a diverse range of glycosidic bonds.

Endophytic fungi are a type of fungi that inhabit plant hosts without causing visible lesions. These microorganisms significantly influence their host plants by promoting nutrient uptake and growth, enhancing stress resistance, defending against pathogen invasion, and regulating secondary metabolism [17]. Recent studies have indicated that these fungi represent a significant source of valuable catalytic enzymes, particularly glycoside hydrolases [18,19], holding great potential for applications in biocatalysis and biotransformation. Additionally, the enzymes produced by these endophytes play crucial ecological roles, such as facilitating colonization, modulating host immunity, enhancing nutrient acquisition, aiding in pathogen defense, regulating secondary metabolism, and maintaining symbiotic balance [20]. In our previous studies, we isolated an endophytic *Chaetomium globosum* DX-THS3 from Dongxiang wild rice (*Oryza rufipogon* Griff.), which exhibited a high abundance of glycoside hydrolases [21,22]. Furthermore, we previously identified a glucuronidase from this fungus that efficiently and specifically converts glycyrrhizic acid, underscoring the strain’s significant potential in the biotransformation of glycosidic compounds. In this study, we report the discovery of a glycoside hydrolase gene, *Xyaf313*, belonging to the GH43 family in the endophytic *C. globosum* DX-THS3. We successfully expressed the recombinant Xyaf313 in *Pichia pastoris* and performed a detailed analysis of its biochemical properties. Furthermore, we explored the enzyme’s potential applications in the biotransformation of ginsenosides. Our findings contribute to the identification of a new class of bifunctional GH43 glycoside hydrolases, highlight the significance of plant endophytic fungi as promising resources for the screening of carbohydrate-decomposing enzymes, and present new candidate enzymes for the biotransformation of ginsenosides.

## 2. Materials and Methods

### 2.1. Strains and Chemical Reagents

*C. globosum* DX-THS3 is a plant endophytic fungus that was isolated and screened from the stems of Dongxiang wild rice in our previous study [20]. This strain has been deposited in the Chinese Archives Culture Collection Center of Wuhan University (Accession No. CCTCCM2016005). In this study, we utilized *P. pastoris* GS115, which was preserved in our laboratory, while the expression plasmid pPIC9K was obtained from the Invitrogen Company in the United States. We sourced restriction enzymes, including EcoRI, NotI, AatII, and SacI, as well as DNA standards and a DNA/protein loading buffer, from Takara (Osaka, Japan). The plasmid extraction kit and Taq Mix polymerase were acquired from Vazyme (Nanjing, China). Additionally, peptone, glucose, yeast, and agar powder were purchased from Aladdin (Shanghai, China).

Ginsenosides Rb_1_, Rb_2_, Rb_3_, Rc, Rd, and Mc were obtained from Chengdu Mansite Biotechnology Co., Ltd. (Chengdu, China) Penicillin (G418), beech wood xylan, sodium carboxymethyl cellulose, methanol, glycerol, cellulose, and avicel were sourced from Shanghai Yuanyang Biotechnology Co., Ltd. (Shanghai, China) Additionally, 4-nitrophenyl-β-D-xyloside (ρNPX), 4-nitrophenyl-β-D-galactopyranoside (ρNPG), and 4-nitrophenyl α-L-arabinofuranoside (ρNPA) were acquired from Beijing Solbaike Biotechnology Co., Ltd. (Beijing, China).

### 2.2. Gene Cloning, Expression, and Purification

Based on the whole-genome sequencing results of strain *C. globosum* DX-THS3 and a comparative analysis using the Carbohydrate-Active Enzyme Database (CAZy), the potential xylosidase gene, *Xyaf313*, was selected and submitted to the China National GeneBank Database (https://db.cngb.org/, Accession No. CNP0006855). An EcoR I restriction site was introduced at the 5′ end of gene *Xyaf313*, while a Not I restriction site was added to the 3′ end. Additionally, six His tags were incorporated before the stop codon to facilitate subsequent protein purification. The synthesis of the gene was performed by Beijing Qingke Biotechnology Co., Ltd., where the synthesized gene sequence was codon-optimized and subsequently cloned into the *P. pastoris* expression vector pPIC9K. A substantial quantity of the recombinant expression vector pPIC9-Xyaf313 plasmid was extracted, linearized via AatII single-enzyme digestion, and the resulting digested product was recovered and transformed into GS115 competent cells using electroporation, followed by spreading on MD plates. After an inverted culture at 28 °C for 2–3 days, individual colonies on the MD plate were selected and transferred to BMGY medium. Cultivation was carried out on a constant-temperature shaker at 28 °C and 220 r/min until the OD_600_ value reached 2–6. The culture was then centrifuged at 5000× *g* for 5 min, the supernatant was discarded, and the cells were resuspended in BMMY medium. Induction and culture were conducted under 0.5% methanol for 5 days, after which the crude enzyme solution was prepared by centrifugation at 3000× *g* and 4 °C for 10 min.

The purification steps for the recombinant protein are as follows. First, place the crude enzyme solution in a magnetic stirring device and gradually add ammonium sulfate powder while stirring until saturation is achieved. This process should be conducted in an ice water bath to prevent temperature fluctuations from affecting the enzyme’s activity and compromising subsequent experiments. Next, transfer the resulting solution to a centrifuge tube and centrifuge it at 10,000× *g* for 15 min at 4 °C to collect the protein precipitate. Dissolve the protein precipitate in 0.1 M PBS (pH 7.4) buffer, and then transfer the enzyme solution to a dialysis bag. Place the bag in a beaker containing 0.1 M PBS (pH 7.4) for dialysis, and refrigerate it at 4 °C overnight. Finally, purify the dialyzed enzyme solution using Thermo Fisher’s HisPur Ni-NTA spin column kit (Waltham, MA, USA) to obtain the recombinant protein Xyaf313. The protein size was determined by SDS-PAGE, and the protein concentration was measured using the Bradford method, with bovine serum albumin serving as the standard.

### 2.3. Enzyme Activity Measurement

To measure the enzyme activity, mix 50 μL of 5 mM ρNPAF with 200 μL of buffer and preheat the mixture at the optimal temperature for 5 min. Subsequently, add 50 μL of the appropriately diluted purified enzyme solution and allow the reaction to proceed under optimal conditions for 10 min. Immediately after the reaction period, terminate the reaction by adding 150 μL of Na_2_CO_3_. Utilize a microplate reader to measure the absorbance of the reaction product at 450 nm, and calculate the concentration of ρNP generated using the ρNP standard curve. Under specified conditions, the amount of enzyme required to hydrolyze 1 μmol of ρNPAF per minute is defined as one unit (U) of arabinofuranosidase activity.

Mix 50 μL of 5 mM ρ-nitrophenyl β-D-xylopyranoside (ρNPX) with 200 μL of buffer and preheat the mixture at the optimal temperature for 5 min. Subsequently, add 50 μL of the appropriately diluted enzyme solution and mix under optimal conditions. After allowing the reaction to proceed for 10 min, immediately add 150 μL of Na_2_CO_3_ to terminate the reaction. Use a microplate reader to measure the absorbance of the reaction product at 450 nm, and calculate the concentration of ρNP generated by the reaction according to the ρNP standard curve. The amount of enzyme required to hydrolyze 1 μmol of ρNPX per minute is defined as 1 xylosidase activity unit (U).

### 2.4. Biochemical Characterization Analysis

The properties of the recombinant Xyaf313 were evaluated in relation to the temperature, pH, organic reagents, and metal ions. First, optimal temperature experiments for arabinofuranosidase and xylosidase activity were conducted across a range of temperatures (30–90 °C). Secondly, the optimal pH of the recombinant Xyaf313 was examined, with values ranging from 3.0 to 10.5. Citrate buffer was used for pH levels of 3.0–6.0, phosphate buffer for pH levels of 6.0–9.0, and glycine–NaOH buffer for pH levels of 9.0–10.5.

Recombinant Xyaf313 was incubated at temperatures of 30, 40, 50, and 60 °C for varying time periods (0, 30, 60, 90, and 120 min), after which the enzyme activity was measured under the optimal reaction conditions, respectively. The activity of arabinofuranosidase or xylosidase at 0 min was defined as 100%. Subsequently, the relative enzyme activity was calculated for other temperatures and time periods, allowing for an analysis of the enzyme’s temperature stability.

We mixed 20 μL of recombinant Xyaf313 with 200 μL of buffer at varying pH values (3.0, 4.0, 5.0, 6.0, 7.0, 8.0, 9.0, and 10.5). We placed the mixtures in a refrigerator at 4 °C and incubated them for 24 h. Subsequently, we measured the enzymatic activity of α-L-arabinofuranosidase and xylosidase under optimal reaction conditions. The activity of untreated recombinant Xyaf313 was defined as 100%, and the relative enzyme activity under different pH treatments was calculated to assess the pH stability of the enzyme.

Recombinant Xyaf313 was incubated with 0.1% and 1% concentrations of various organic solvents, including methanol, Triton X-100, Tween, glycerol, β-mercaptoethanol, EDTA, and SDS, for 1 h at 4 °C to assess the relative activity. Each experiment was conducted in triplicate. Recombinant Xyaf313 was incubated with 1 mmol/L and 5 mmol/L solutions of Na^+^, NH_4_^+^, K^+^, Al^3+^, Zn^2+^, Fe^3+^, Cu^2+^, Mn^2+^, Co^2+^, Mg^2+^, Ni^2+^, and Fe^3+^ for 1 h at 4 °C to assess the relative activity. Each experiment was conducted in triplicate.

### 2.5. Substrate Specificity and Enzymatic Kinetics Analysis

In this study, various natural and synthetic substrates were utilized to investigate the substrate specificity of recombinant Xyaf313. The natural substrates included beech wood xylan, microcrystalline cellulose, and carboxymethyl cellulose, while the synthetic substrates comprised ρ-nitrophenyl-β-D-xyloside (ρNPX), ρ-nitrophenyl-α-L-arabinofuranoside (ρNPAF), ρ-nitrophenyl-β-D-cyanoside, ρ-nitrophenyl-β-D-glucoside (ρNPG), and ρ-nitrophenyl-β-D-glucuronide (ρNPG). These substrates were employed to assess the substrate specificity of xylanases. Experiments were conducted under optimal conditions, with the maximum enzyme activity defined as 100%, allowing for the calculation of the relative enzyme activity of the other substrates. Each experimental group was replicated three times.

The K_m_, V_max_, and K_cat_ values for purified recombinant Xyaf313 were determined from Michaelis–Menten plots using the nonlinear regression software GraphPad (Prism 5.0). To prepare the substrate solutions, we created 0.5, 1.0, 1.5, 2.0, 2.5, 3.0, 3.5, 4.0, 4.5, and 5.0 mM concentrations of either ρNPAF or ρNPX, respectively. Subsequently, we measured the amount of ρNP produced by the reaction under optimal conditions. Each experimental group was replicated three times. By plotting the substrate concentration on the x-axis and the reaction velocity on the y-axis, we utilized nonlinear curve fitting in the Origin software (origin 2021) to generate a Michaelis–Menten plot, allowing for the calculation of the maximum reaction velocity (V_max_) and K_m_ values for recombinant Xyaf313.

### 2.6. Biotransformation of Ginsenosides

Accurately weigh 5 mg of the ginsenoside Rb_1_, Rb_2_, Rb_3_, and Rc standards and dissolve them in 5 mL of PBS buffer to prepare a substrate with a concentration of 1 mg/mL. Combine 500 μL of the substrate with 500 μL of the purified recombinant Xyaf313 enzyme solution and allow the reaction to proceed at 37 °C for 24 h. To terminate the reaction, boil the mixture for 5 min and then centrifuge the reaction product at 8000 rpm for 5 min for subsequent analysis. After collecting the transformation products, extract and stratify them with equal volumes of n-butanol, separating the upper n-butanol phase. This phase is then placed in a rotary evaporator and evaporated until it forms a powder. The resulting powder is dissolved in 1.5 mL of chromatography-grade methanol, filtered through a 0.25 μm organic filter, and placed in a liquid-phase vial for high-performance liquid chromatography detection.

The filling material of the chromatographic column utilized in the high-performance liquid chromatography (HPLC) analysis was octadecylsilane-bonded silica gel. The column was characterized by a length of 250 mm, an inner diameter of 4.6 mm, and a particle size of 5 μm. The mobile phases employed included water as mobile phase A and acetonitrile as mobile phase B, which together facilitate gradient elution. The column temperature was maintained at 30 °C, with the detection wavelength set at 203 nm.

## 3. Results and Discussion

### 3.1. Xyaf313 Gene Sequence Analysis and Expression

**Figure 1:**
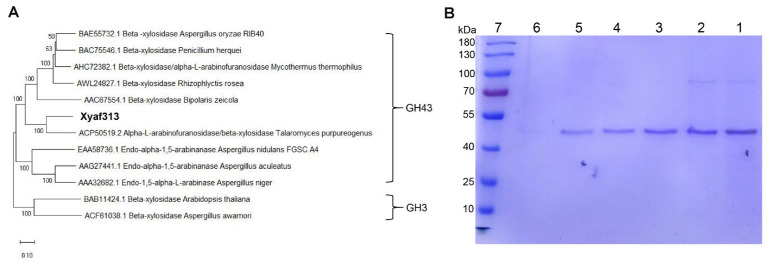
The genetic analysis and expression reorganization of Xyaf313 were conducted. Based on the phylogenetic tree constructed from amino acid sequences (**A**), the SDS-PAGE analysis of Xyaf313 was conducted (**B**). The SDS-PAGE gel lanes are organized as follows: lanes 1–2 display the induced crude enzyme solution, lanes 3–5 represent purified Xyaf313, lane 6 represents the uninduced crude enzyme solution, and lane 7 displays the protein marker.

In our previous research, we identified an endophytic strain of *C. globosum* DX-THS3 that possesses a rich array of glycoside hydrolases, characterized by an efficient lignocellulose-degrading enzyme system [18,19]. The glycosidase Xyaf313, investigated in this study, is derived from this endophytic *C. globosum* DX-THS3. The gene encoding this enzyme contains 1380 base pairs and translates into a protein of 460 amino acids, with a theoretical molecular weight of approximately 50 kDa. Phylogenetic analysis based on the amino acid sequence indicates that Xyaf313 belongs to the glycoside hydrolase family 43 (GH43) and exhibits the highest similarity to α-L-arabinofuranosidase/β-D-xylosidase from *Talaromyces purpureogenus*, clustering within the same branch (Figure 1A). This suggests that Xyaf313 is a potential bifunctional glycoside hydrolase. To our knowledge, the CAZy database lists 239 functionally characterized glycosidases in the GH43 family, predominantly from bacterial sources; only 39 have been identified from fungi and none from *C. globosum*. Many studies have indicated that GH43 family glycosidases are prevalent in lignocellulose-degrading microorganisms, as these enzymes are crucial components of the hemicellulase system [2,3,4]. Our findings support this assertion, as the host *C. globosum* DX-THS3 also demonstrates efficient lignocellulose degradation capabilities. Furthermore, this work may provide new candidate strains for the discovery and screening of GH43 family glycosidases. Subsequently, we induced the expression of the Xyaf313 gene in *P. pastoris* and purified the product using a Ni^2+^ affinity column. SDS-PAGE analysis confirmed the successful isolation of the soluble Xyaf313 (Figure 1B). Notably, the observed molecular weight is slightly greater than the theoretical value (Figure 1B), which may indicate the occurrence of eukaryotic glycosylation modifications during expression.

### 3.2. Enzyme Characteristics

#### 3.2.1. Substrate Specificity Analysis

The phylogenetic tree analysis indicated that Xyaf313 belongs to the GH43 family and exhibits the greatest similarity to the bifunctional enzyme derived from *T. purpureogenus* (Figure 1A), suggesting that Xyaf313 may also function as a multifunctional enzyme. To verify this hypothesis, we evaluated the substrate hydrolysis capability of the recombinant Xyaf313. As shown in Table 1, our results demonstrate that Xyaf313 can significantly hydrolyze the substrates ρNPAF and ρNPX, confirming that Xyaf313 possesses bifunctional enzyme activity with glycosidase activity. Additionally, the results indicate that Xyaf313 has a strong effect on ρ-nitrophenyl-β-D-glucuronide, while other substrates, including ρ-nitrophenyl-β-D-glucopyranoside, ρ-nitrophenyl-β-D-galactopyranoside, ρ-nitrophenyl-β-D-mannopyranoside, ρ-nitrophenyl-α-L-rhamnopyranoside, and ρ-nitrophenyl-α-L-arabinopyranoside, exhibit only slight hydrolysis activity (Table 1). However, Xyaf313 does not demonstrate hydrolytic activity towards complex polysaccharide molecules such as cellulose and xylan. Glycoside hydrolases from the GH43 family predominantly exhibit arabinofuranosidase and xylosidase activity, which aligns with our findings. Notably, some studies have reported that certain members of the GH43 family possess the capability to hydrolyze multiple glycosidic bonds simultaneously. For instance, PphXyl43B from *Paenibacillus physcomitrellae* demonstrates both β-xylosidase and α-L-arabinofuranosidase activity [23]. Additionally, Viborg et al. [15] identified a GH43 glycosidase, BXA43, with bifunctional β-D-xylosidase and α-L-arabinofuranosidase activity. However, instances of GH43 family glycosidases exhibiting bifunctional activity remain scarce, with most identified to date being of bacterial origin. In contrast, the enzyme Xyaf313 found in this study not only displays clear dual functions as an arabinofuranosidase and xylosidase but also originates from a plant endophytic fungus. Importantly, numerous recent studies have demonstrated that plant endophytic fungi serve as a significant source of catalytic enzymes, particularly glycoside hydrolases [18,19,24], and our results further substantiate this assertion.

Given that Xyaf313 demonstrates significant arabinofuranosidase and xylosidase activity, our subsequent studies will focus on the enzymatic properties associated with these two types of activity. Our findings indicate that when Xyaf313 exhibits α-L-arabinofuranoside activity, its specific enzyme activity measures 23.96 U/mg, with a K_m_ of 5.65 mM, V_max_ of 486.47 μmol min^−1^ mg^−1^, and K_cat_ of 64.53 S^−1^. In contrast, when Xyaf313 displays xylosidase activity, the specific enzyme activity is 23.24 U/mg, with a K_m_ of 1.77 mM, V_max_ of 542.65 μmol min^−1^ mg^−1^, and K_cat_ of 71.90 S^−1^ (Table 2).

#### 3.2.2. Biochemical Characteristics of Xyaf313 with α-L-Arabinofuranoside Activity

**Figure 2:**
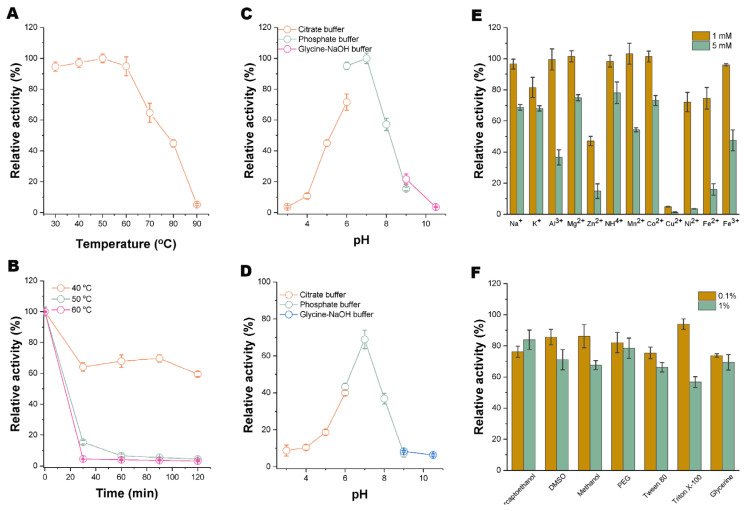
Biochemical characterization of recombinant Xyaf313, when exhibiting α-L-arabinofuranosidase activity. (**A**) illustrates the effect of the temperature on recombinant Xyaf313, while (**B**) provides an analysis of its temperature stability. (**C**) shows the impact of the pH on recombinant Xyaf313, and (**D**) details the pH stability analysis. Additionally, (**E**) shows the influence of metal ions on recombinant Xyaf313, and (**F**) shows the effects of various chemical reagents on the enzyme.

We first analyzed the biochemical characteristics of Xyaf313 in relation to its arabinofuranosidase activity. As illustrated in Figure 2A, Xyaf313 exhibits peak activity at 50 °C. However, when the temperature exceeds 60 °C, there is a rapid decline in residual enzyme activity, which aligns with the characteristics typical of a mesophilic enzyme. Notably, Xyaf313 demonstrates significant catalytic activity, with relative enzyme activity exceeding 90%, at room temperature (~30 °C). Furthermore, the temperature stability analysis indicated that Xyaf313 maintains good stability at temperatures of 40 °C or lower. After a 120 min incubation period, its relative residual enzyme activity remains above 60% (Figure 2B). These findings suggest that the enzyme has promising application potential and can mitigate the cost issues associated with harsh reaction conditions, such as elevated temperatures.

Subsequently, we analyzed the effect of the pH on recombinant Xyaf313. The results indicated (Figure 2C) that the optimal pH for this enzyme is 7, and it is sensitive to both acidic and alkaline conditions, which aligns with the characteristics of most glycosidases in the GH43 family. The pH stability assessment also revealed (Figure 2D) that the enzyme remains relatively stable under neutral conditions. After a 24 h incubation period within the pH range of 6 to 8, its relative residual enzyme activity exceeds 40%. However, its activity is significantly affected in excessively acidic or alkaline environments.

In addition, we analyzed the effects of various metal ions and common chemical reagents on the arabinofuranosidase activity of Xyaf313. The results indicate that, at lower concentrations, most metal ions, with the exception of Cu^2+^ and Zn^2+^, have a minimal impact on the activity of Xyaf313. However, when the concentration is increased to 5 mM, most metal ions significantly affect the enzyme’s activity, particularly Cu^2+^, which almost completely inhibits the activity of Xyaf313 (Figure 2E). Conversely, while common chemical reagents also influence the activity of Xyaf313, the relative residual enzyme activity in the presence of most chemical reagents remains at 60–80% (Figure 2F). This suggests that Xyaf313 demonstrates greater tolerance to chemical reagents.

#### 3.2.3. Biochemical Characteristics of Xyaf313 with β-D-Xylosidase Activity

**Figure 3:**
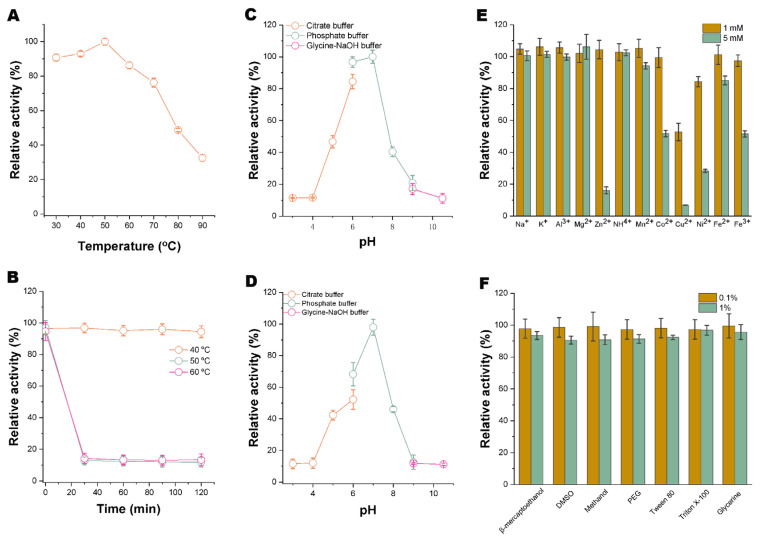
Biochemical characterization of recombinant Xyaf313, when exhibiting β-D-xylosidase activity. (**A**) illustrates the effect of the temperature on recombinant Xyaf313, while (**B**) provides an analysis of its temperature stability. (**C**) shows the impact of the pH on recombinant Xyaf313, and (**D**) details the pH stability analysis. Additionally, (**E**) shows the influence of metal ions on recombinant Xyaf313, and (**F**) shows the effects of chemical reagents on the β-D-xylosidase activity of Xyaf313.

Furthermore, we analyzed the biochemical characteristics of Xyaf313 in relation to its xylosidase activity. As illustrated in Figure 3A, the enzyme activity remains elevated within the temperature range of 30–70 °C, with the highest activity observed at 50 °C. However, when the temperature exceeds 70 °C, there is a significant decrease in enzyme activity. The temperature stability analysis indicates that the xylosidase activity of Xyaf313 exhibits excellent stability at temperatures below 40 °C, as its residual enzyme activity does not significantly decline after 120 min of incubation (Figure 3B). In contrast, the stability diminishes notably at temperatures exceeding 50 °C.

The influence of the pH on Xyaf313’s xylosidase activity exhibits a trend similar to that observed when Xyaf313 demonstrates α-L-arabinofuranosidase activity (Figure 3C,D). The optimal reaction pH for both types of activity is 7.0, and the enzyme is sensitive to variations in acid–base environments (Figure 3C). In comparison to the α-L-arabinofuranosidase activity, the impact of metal ions on Xyaf313’s xylosidase activity is generally less pronounced, with the exceptions of Cu^2+^ and elevated concentrations of Ni^2+^ and Zn^2+^ (Figure 3E). Notably, neither 0.1% nor 1% concentrations of common chemical reagents exert a significant effect on Xyaf313’s xylosidase activity (Figure 3F). Overall, when Xyaf313 exhibits xylosidase activity, it demonstrates greater resistance to metal ions and chemical reagents than when it exhibits α-L-arabinofuranosidase activity.

### 3.3. Transformation of Ginsenosides to Selectively Produce Ginsenoside Rd by Xyaf313

**Figure 4:**
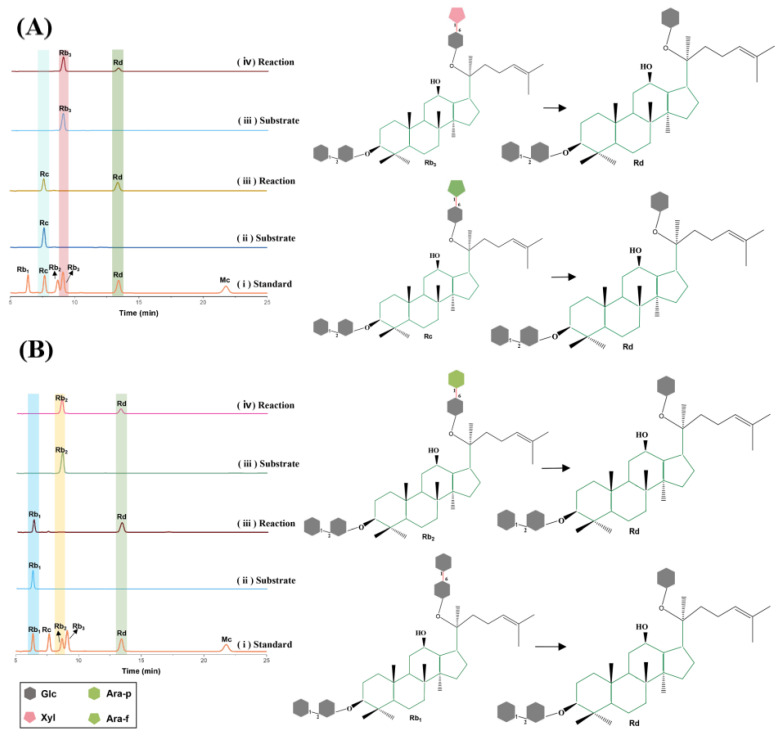
The analysis of recombinant Xyaf313 focusing on its ability to transform ginsenosides. (**A**) represents the product obtained from the high-efficiency liquid chromatography analysis of ginsenosides Rb_3_ and Rc transformed by Xyaf313. (**B**) shows the product resulting from the high-efficiency liquid-phase analysis of reorganized Xyaf313, which transforms ginsenosides Rb_1_ and Rb_2_.

Ginseng has been utilized as a nourishing and strengthening medicinal material in China and other Asian countries for thousands of years [25]. Ginsenosides are the primary components responsible for the various pharmacological and biological properties of ginseng [26]. Research conducted over the past decade has revealed that different ginsenosides possess significant medicinal value and healthcare functions [26], particularly in terms of immune regulation, therapeutic potential for diabetes [27], anti-tumor effects [28], and anti-aging properties [29]. The structures of ginsenosides include common sugar groups such as β-glucose, L-arabinose, D-xylose, and L-rhamnose, which necessitate the collaborative action of various enzymes to achieve biotransformation [26]. This requirement not only complicates the operational process but also increases the enzyme costs [8]. The bifunctional enzyme Xyaf313, identified in this study, exhibits both xylosidase and arabinofuranosidase activity, indicating its considerable potential for application in the field of ginsenoside biotransformation. To investigate the biotransformation function of Xyaf313 regarding ginsenosides, we selected Rb_3_ and Rc, which contain xylose and arabinofuranosyl groups, respectively, for a transformation investigation. After a 24 h reaction period, it was observed that the products obtained from the Xyaf313-catalyzed transformations of Rb_3_ and Rc were exclusively Rd, with no additional products detected (Figure 4A). This finding aligns with the β-glucosidase and α-L-arabinofuranoside activity exhibited by this enzyme. Notably, in addition to catalyzing Rb_3_ and Rc, Xyaf313 is also capable of converting Rb_2_ and Rb_1_, which contain arabinopyranosyl and glucose groups, into Rd (Figure 4B). Consequently, our results indicate that Xyaf313 can recognize a diverse range of ginsenoside substrates and specifically produce Rd. Its transformation characteristics reveal that it can selectively hydrolyze the 1–6 glycosidic bonds on the exterior of ginsenoside C-20, while the 1–2 glycosidic bonds remain inactive. Furthermore, Xyaf313 can catalyze various types of glycosidic bonds, including Glu-Ara (furan), Glu-Ara (pyran), Glu-Xyl, and Glu-Glu.

Currently, a considerable number of catalytic enzymes capable of transforming ginsenosides have been identified [10,30]. These enzymes are predominantly β-glucosidases, with the majority being derived from microorganisms [10]. Due to the intricate sugar structures of ginsenosides, glycoside hydrolases that act upon them face challenges, including diverse conversion pathways and the generation of complex products. For instance, Renchinkhand et al. [31] isolated a strain of *Lentilactobacillus buchneri* from Korean fermented plant foods. This strain produces β-glucosidase, which hydrolyzes ginsenoside Rb_1_ to generate rare ginsenosides Rd and Rg_3_. Similarly, Upadhyaya et al. [12] purified a novel hydrolyzing enzyme from *Armillaria mycelium*, designated BG-1, which can simultaneously hydrolyze the α-(1→6)-arabinofuranosyl bond at the C-20 position of Rc and the external β-arabinose bond at the C-3 position of the Rc (1→2)-glycosidic bond, resulting in the production of Rd and C-Mc1. Additionally, Zeng et al. [11] cloned the β-glucosidase Tsbgl from *Thermoclostridium stercorarium*, which is capable of hydrolyzing all outer glucose groups of protopanaxadiol-type ginsenosides Rb_1_, Rb_2_, Rc, and Rb_3_, as well as protopanaxadiol-type ginsenosides Re and Rg_1_, through various cleavage pathways, yielding a variety of rare ginsenosides. Notably, the lack of specificity in the current methods results in challenges during subsequent separation processes and leads to low yields of the target rare ginsenosides, significantly hindering the application potential of these compounds. On the other hand, the recently discovered α-L-araoinopyranosidase is often limited to converting only specific ginsenosides [13,14], which poses additional challenges for the application of this enzyme type. In this study, we identified the bifunctional glycosidase Xyaf313, which demonstrates the ability to hydrolyze various ginsenoside substrates, indicating a wide range of substrate adaptability. Intriguingly, our study revealed that Xyaf313 not only hydrolyzes the xylosyl and arabinofuranosyl moieties of ginsenosides Rb1 and Rc—consistent with its anticipated substrate scope—but also exhibits unexpected activity toward the arabinopyranosyl and outer glucosyl groups of ginsenosides Rb_2_ and Rb_1_, yielding ginsenoside Rd as the terminal transformation product (Figure 4). Notably, however, the enzyme failed to cleave the inner glucosyl residue of Rd, suggesting strict regiospecificity. This distinct catalytic behavior highlights Xyaf313’s ability to selectively hydrolyze glycosidic bonds at the C-20 position in protopanaxadiol-type ginsenosides, a feature accompanied by pronounced stereoselectivity. Such dual specificity and stereochemical discrimination are exceptionally rare among characterized multifunctional ginsenoside-transforming enzymes [32], positioning Xyaf313 as a unique biocatalytic tool for the targeted structural modification of triterpenoid glycosides. Notably, its enzymatic activity exclusively produces ginsenoside Rd, which is highly significant for the targeted large-scale preparation of this compound. Previous studies have shown that ginsenoside Rd exhibits anti-aging [26], neuroprotective [33,34], immunity-enhancing [26], and anti-cancer properties [35]. Furthermore, ginsenoside Rd serves as a precursor for many rare ginsenosides. When combined with β-glucosidase, it can be further hydrolyzed to yield rare ginsenosides with enhanced activity, such as CK and Rg_3_ [10,36]. In conclusion, our findings introduce a class of glycoside hydrolases capable of hydrolyzing a variety of common ginsenosides for the targeted production of Rd, while also highlighting the application potential of GH43 glycosidases in the field of ginsenoside biotransformation.

## 4. Conclusions

In this study, we identified a bifunctional glycoside hydrolase from the GH43 family, derived from the plant endophytic fungus *C. globosum* DX-THS3. The enzyme, designated Xyaf313, exhibits α-L-arabinofuranoside activity, with specific enzyme activity of 23.96 U/mg, a K_m_ of 5.65 mM, a V_max_ of 486.47 μmol min^−1^ mg^−1^, and a K_cat_ of 64.53 S^−1^, with optimal activity at 50 °C and a pH of 7. In addition, when exhibiting β-D-xylosidase activity, Xyaf313 shows specific enzyme activity of 23.24 U/mg, a K_m_ of 1.77 mM, a V_max_ of 542.65 μmol min^−1^ mg^−1^, and a K_cat_ of 71.90 S^−1^, also with optimal conditions at 50 °C and pH 7. Furthermore, both types of activity of Xyaf313 demonstrate good resistance to common metal ions and chemical reagents. The results from ginsenoside conversion tests indicate that Xyaf313 can convert various ginsenoside substrates, including ginsenosides Rc, Rb_1_, Rb_2_, and Rb_3_, yielding a specific product, ginsenoside Rd. In summary, our findings reveal a novel bifunctional GH43 family glycosidase with both α-L-arabinofuranoside and xylosidase activity, capable of recognizing a range of common ginsenosides and converting them into ginsenoside Rd, highlighting its potential applications in the medical field.

## Figures and Tables

**Table 1:** Substrate specificity of glycosidase Xyaf313.

Substrate	Relative Activity (%)
ρ-Nitrophenyl-α-L-arabinofuranoside	100
ρ-Nitrophenyl-β-D-xylopyranoside	97 ± 3.29
p-Nitrophenyl β-D-glucuronide	2 ± 0.46
ρ-Nitrophenyl-β-D-glucopyranoside	1 ± 0.15
ρ-Nitrophenyl-β-D-galactopyranoside	1 ± 0.23
ρ-Nitrophenyl-β-D-mannopyranoside	3 ± 0.56
ρ-Nitrophenyl-α-L-rhamnopyranoside	2 ± 0.32
ρ-Nitrophenyl-α-L-arabinopyranoside	4 ± 0.68
CMC-Na	ND
Cellulose	ND
Avicel	ND
Xylan (Beech wood)	ND

ND indicates that the enzymatic activity was not detected.

**Table 2:** The kinetic parameters of xylanase Xyaf313.

Activity	K_m_ (mM)	V_max_ (μmol min^−1^ mg^−1^)	k_cat_ (S^−1^)	k_cat_/K_m_ (S^−1^ mM^−1^)
Arabinofuranosidase	5.65 ± 1.67	486.47 ± 2.33	64.53 ± 3.93	11.42 ± 3.44
Beta-Xylanase	1.77 ± 1.27	542.65 ± 6.75	71.90 ± 3.95	40.62 ± 5.63

## Data Availability

The original contributions presented in this study are included in the article. Further inquiries can be directed to the corresponding authors.

## Abbreviations

The following abbreviations are used in this manuscript:

BGL	β-Glucosidase
GH43	Glycoside hydrolase 43
ρNPX	ρ-Nitrophenyl-β-D-xyloside
ρNPG	4-Nitrophenyl-β-D-galactopyranoside
ρNPAF	4-Nitrophenyl α-L-arabinofuranoside
